# All-cause and Cause-specific Mortality in People With HIV in Italy in 1997–2022: Data From the Icona Cohort

**DOI:** 10.1093/ofid/ofaf455

**Published:** 2025-08-02

**Authors:** Andrea Giacomelli, Simone Lanini, Andrea De Vito, Sara De Benedittis, Maria Mazzitelli, Manuela Ceccarelli, Roberta Gagliardini, Giordano Madeddu, Eugenia Quiros-Roldan, Enrico Girardi, Davide Checchi, Giuseppe Lapadula, Carlo Tascini, Alessandro Tavelli, Andrea Antinori, Antonella d'Arminio Monforte

**Affiliations:** III Infectious Diseases Unit, ASST Fatebenefratelli Sacco, Milan, Italy; Department of Biomedical and Clinical Sciences, Università Degli Studi di Milano, Milan, Italy; Dipartimento di Medicina dell'Università di Udine, U.O. Malattie Infettive, Università di Udine e Azienda Sanitaria Universitaria Integrata di Udine, Udine, Italy; Unit of Infectious Disease, Department of Medicine, Surgery and Pharmacy, University of Sassari, Sassari, Italy; ICONA Foundation, Milan, Italy; Infectious and Tropical Diseases Unit, Padua University Hospital, Padua, Italy; Unit of Infectious Diseases, Department of Medicine and Surgery, “Kore” University of Enna, Enna, Italy; Clinical Infectious Diseases Department, National Institute for Infectious Diseases Lazzaro Spallanzani IRCCS, Rome, Italy; Unit of Infectious Disease, Department of Medicine, Surgery and Pharmacy, University of Sassari, Sassari, Italy; Department of Clinical and Experimental Sciences, Unit of Infectious and Tropical Diseases, University of Brescia and ASST Spedali Civili di Brescia, Brescia, Italy; Scientific Direction, National Institute for Infectious Diseases, Lazzaro Spallanzani IRCCS, Rome, Italy; Department of Infectious Diseases, Fondazione Policlinico Tor Vergata, Rome, Italy; Department of Infectious Diseases, IRCCS San Gerardo dei Tintori, University of Milano Bicocca, Monza, Italy; Dipartimento di Medicina dell'Università di Udine, U.O. Malattie Infettive, Università di Udine e Azienda Sanitaria Universitaria Integrata di Udine, Udine, Italy; ICONA Foundation, Milan, Italy; National PhD Programme in One Health Approaches to Infectious Diseases and Life Science Research, Department of Public Health, Experimental and Forensic Medicine, University of Pavia, Pavia, Italy; Clinical Infectious Diseases Department, National Institute for Infectious Diseases Lazzaro Spallanzani IRCCS, Rome, Italy; ICONA Foundation, Milan, Italy

**Keywords:** AIDS, death, late presentation, mortality, universal antiretroviral therapy

## Abstract

**Background:**

Understanding the evolution and dynamics of deaths in people with HIV (PWH) is crucial to tailor interventions aiming at improving PWH long-term well-being. We aimed to assess all-cause and cause-specific mortality in PWH in Italy.

**Methods:**

PWH enrolled before antiretroviral start from Icona cohort (78 Italian HIV clinics) between 1997 and 2021 (last observation December 2022) were included. Mortality was reported as incidence rate per 100 person-years of follow-up (PYFU). The mortality incidence rate according to calendar period was estimated by Poisson regression model.

**Results:**

Overall, 17,006 PWH were included of whom 1584 (9.31%) died. The highest mortality rates were observed during the earliest calendar periods, with 2.67 (95% CI: 2.19–3.25) and 1.93 (95% CI: 1.67–2.22) deaths per 100 PYFU in 1997–1998 and 1999–2001, respectively. After 2010, mortality rates fell below 1 per 100 PYFU, reaching 0.74 (95% CI: 0.65–0.84) and 0.71 (95% CI: 0.63–0.80) in 2017–2019 and 2020–2022, respectively. A significant drop was observed for AIDS-related mortality in the first two periods from 1.45 (95% CI: 1.11–1.90) in 1997–1998 to 0.78 (95% CI: 0.62–0.97) deaths per 100 PYFU in 1999–2001. AIDS-related mortality continued to decrease in the subsequent years, with the lowest rate observed in the last two calendar periods: 0.10 (95% CI: 0.07–0.14) deaths per 100 person-years in 2017–2019 and 0.10 (95% CI: 0.08–0.15) deaths per 100 person-years in 2020–2022.

**Conclusions:**

All-cause mortality in PWH in Italy significantly decreased over time, mainly for a reduction in AIDS-related mortality.

## Teaser

All-cause mortality among people with HIV in Italy has significantly decreased over time, primarily due to a reduction in AIDS-related deaths. Our findings highlight persistent disparities, with higher mortality risks still observed in individuals presenting with AIDS in recent years.

Assessing mortality and causes of death among people with HIV (PWH) is essential for monitoring the long-term impact of HIV and antiretroviral therapy (ART) on survival outcomes, as well as for tailoring interventions aiming at improving the long-term PWH well-being [1]. Despite the significant reduction in HIV-related mortality achieved by ART implementation [2], PWH still face a higher risk of premature death compared to the general population [3]. Even though ART has been extensively offered after 2015, this risk still persists, especially for people diagnosed with HIV and low CD4+ T cell counts [3, 4]. This excess risk for mortality is partly attributed to HIV-associated chronic immune activation and inflammation, despite achievement of a durable virological suppression [5].

Research from European cohorts demonstrated a shift in the primary causes of death among PWH in the ART era. Indeed, there has been a decline in AIDS-related deaths and a relative increase in deaths caused by non-AIDS-defining conditions, including cardiovascular disease, liver disease, non-HIV-related cancers, and aging-related comorbidities [6–8]. Moreover, studies such as the one performed by Antiretroviral Therapy Cohort Collaboration [6], EuroSIDA [9, 10], and RESPOND [11] have highlighted the growing contribution of non-HIV-related causes to overall mortality.

Italy's HIV epidemic initially affected people who inject drugs (PWID) but has since shifted predominantly to sexual transmission [12, 13]. Despite free access to care, testing, and treatment, late presentation remains a major issue, with up to 60% of PWH diagnosed late in 2023 [14–16]. Late presenters experience the poorest clinical outcomes [17]. While Italy has an effective HIV surveillance network, there is a significant gap in data on long-term outcomes and mortality, which is crucial for evaluating the impact of the disease and interventions. This study aimed at assessing all-cause and cause-specific mortality in PWH within the Icona cohort from 1997 to 2022.

## METHODS

### Study Design

Icona is a nationwide multicenter observational prospective cohort, established in 1997, including adult PWH who are ART-naïve at the time of enrollment in 91 Italian infectious diseases Centers. To date, this is the largest and most representative cohort of PWH naïve to ART in Italy. Further details about the cohort are available elsewhere [12].

The study proposal was shared with PWH representatives at the annual Icona meeting (2024). The reporting of the study follows the Strengthening the reporting of observational studies in epidemiology guidelines.

### Setting

The present study was conducted in 78 Italian HIV clinics participating in the Icona cohort. Thirteen of the 91 Italian infectious diseases centers ever involved in the Icona cohort were unable to ensure adequate people follow-up (defined as a proportion of drop out >25%) or assess people’s vital status through official regional and national administrative registries, thus were excluded from the present analysis.

### Participants

We included all consecutive PWH included in the Icona cohort from January 1, 1997 to December 31, 2021, who had an available measure of CD4+ T cell and at least one available follow-up after enrollment, and assured adequate follow-up. PWH have been followed-up until death, administrative censoring (discontinuation from Icona cohort participation either by PWH choice or recruiting center), or December 31, 2022.

### Data Collection, Variables, and Study Definitions

Data of the Icona cohort were collected in an electronic eCRF (https://www.icona.org/). The variables considered for the present analysis were calendar period of enrollment-up in cohort (1997–1998, 1999–2001, 2002–2004, 2005–2007, 2008–2010, 2011–2013, 2014–2016, 2017–2019, and 2020–2022), age (in years), sex assigned at birth (male and female), place of birth (Italian and foreign-born), risk factor for HIV acquisition [men having sex with men (MSM), heterosexual contact (HE), PWID, other], CD4+ T cell count at enrollment (<200, 200–350, 350–500, and >500 cell/µL), late presentation (CD4+ T cell count <350 cell/µL and/or AIDS), AIDS events at enrollment (yes and no), hepatitis C serology (positive and negative).

All deaths were included in the analysis, regardless of available details, including cases without a confirmed date. In such cases, death was assumed to have occurred 6 months after the last recorded data point. Vital status was verified using official regional and national administrative registries, though these lacked information on the cause of death. For cases without an ascertained cause, two independent investigators (A.G. and A.D.V.) reconstructed a narrative based on data from the Icona dataset, incorporating clinical, laboratory, demographic, and behavioral information. Queries were directed to HIV physicians for missing details, and in cases of ambiguity or disagreement, a third senior expert (A.D.M.) conducted a blind review [7, 8].

Causes of death were classified as AIDS-related, non-AIDS-defining cancer-related, cardiovascular disease-related, liver-related, and other non-AIDS/non-cancer-related causes. Cases with insufficient information were categorized as “unknown.” AIDS-related deaths were defined based on the presence of a serious AIDS-defining condition, a CD4+ T cell count below 100 cells/µL within one year of death (or within 18 months if not receiving ART), and a recorded AIDS-related cause of death in the database [18, 19]. For cause-specific incidence analyses, deaths were grouped into three broad categories: AIDS-related, non-AIDS-related, and unknown.

### Outcomes

The primary outcome of this study was all-cause mortality, defined as any death occurring from the date of enrollment to the end of follow-up.

The secondary outcome was cause-specific mortality. Specifically, the causes of death were further regrouped into: AIDS-related, non-AIDS-related (including cardiovascular disease, non-AIDS-defining cancers, liver disease, and other non-AIDS causes) and unknown.

### Statistical Analysis

A descriptive statistical analysis was conducted based on the year of enrollment. Crude all-cause and cause-specific mortality rates were reported as incidence rates per 100 person-years of follow-up (PYFU). Time was categorized into 9 calendar periods (1997–1998, 1999–2001, 2002–2004, 2005–2007, 2008–2010, 2011–2013, 2014–2016, 2017–2019, and 2020–2022). Analyses were performed using Poisson regression with robust correction for repeated measures over time. All-cause mortality rates were provided as both unadjusted and adjusted estimates (sex- and age-adjusted). Mortality rate estimates were further stratified by specific exposures: (A) sex (adjusted for current age), (B) age at enrollment(<45 or ≥45 years, based on the mean age at early ICONA enrollment) [12, 13] (adjusted for sex), (C) transmission route (MSM, PWID, HE, other) (adjusted for current age), (D) CD4+ T cell count at enrollment (<350 or ≥350 cells/µL) (adjusted for age and sex), (E) AIDS-defining condition at enrollment (adjusted for age and sex), (F) HCV serology (adjusted for age and sex), and (G) HBsAg positivity (adjusted for age and sex). All models (A–G) included vital status at follow-up as the dependent variable (binary), time as a 9-level categorical variable, and age and/or sex as confounders, along with the specific predictor under assessment. A full categorical-to-categorical interaction between each predictor and time was incorporated. Mortality estimates were presented with robust 95% confidence intervals (CI), and associations were expressed as rate ratios.

## RESULTS

### Characteristics of the Cohort and Enrolled People With HIV

During the study period, 17 006 PWH were included in the analysis (Supplementary Figure 1), contributing to 155 279 PYFU. The characteristics of PWH at enrollment across different calendar periods are summarized in Table 1. The mean age at enrollment increased significantly, from 35.6 (95% CI 35.2–35.9) years in 1997–1998 to 41.7 (95% CI 41–42.3) years in 2020–2022. Also, the proportion of male rose from 68.6% in 1997–1998 to 84.1% in 2020–2022.

**Table 1.** Characteristics of People With HIV Newly Enrolled in the Cohort According to the Calendar Period

Characteristics		Calendar Period								
	Overall	1997–1998	1999–2001	2002–2004	2005–2007	2008–2010	2011–2013	2014–2016	2017–2019	2020–2022
**Number of enrolled**	17 006	3355	957	791	513	1583	2759	3208	2686	1154
**Male sex assigned at birth, %**	77.6%	68.6%	69.6%	69.2%	75.6%	80.9%	80%	81.3%	82.9%	84.1%
**Age at enrollment, mean (95% CI)**	38.7 (38.5–38.8)	35.6 (35.2–35.9)	36.5 (35.9–37.2)	37.8 (37.1–38.6)	38.4 (37.5–39.3)	39 (38.5–39.5)	39.1 (38.7–39.5)	39.6 (39.3–40)	40.5 (40.1–40.9)	41.7 (41–42.3)
**Age strata,%**										
** <45**	75.8%	90.6%	86.3%	83.6%	77.6%	75.7%	72.7%	70.3%	66%	63.3%
** >45**	24.2%	9.4%	13.7%	16.4%	22.4%	24.3%	27.3%	29.7%	34%	36.7%
**Italian, %**	81.4%	95.1%	89.9%	88.7%	85%	83.3%	77.4%	75.5%	72.5%	72.8%
**Mode of HIV acquisition, %**										
** HE**	37.7%	32.1%	43.3%	43.6%	40.7%	39.9%	40.6%	36.4%	37.6%	38.2%
** MSM**	40.2%	17%	22.5%	28.7%	39%	48.1%	45%	51.7%	51.9%	48.3%
** PWID**	16.5%	47.1%	29.5%	19.1%	13.7%	7.6%	7.7%	5.6%	5.8%	4.6%
** Other**	5.6%	3.8%	4.8%	8.6%	6.6%	4.5%	6.5%	6.3%	4.7%	8.9%
**CD4 at enrollment**										
** Mean (95% CI)**	418 (413–422)	481 (471–491)	440 (420–458)	434 (412–454)	414 (388–440)	439 (425–453)	411 (400–422)	404 (393–413)	367 (355–378)	352 (333–368)
** >500 cell/µL, %**	35.1%	44.5%	39.4%	37.7%	33.8%	36.5%	33.4%	33.3%	28.2%	26.6%
** 350–499 cell/µL, %**	20.7%	21.5%	19.1%	20.2%	20.5%	22.7%	22.1%	20.3%	18.9%	18.9%
** 200–349 cell/µL, %**	18.2%	14.8%	16%	18%	20.1%	19.7%	19.1%	18.3%	20.2%	19.6%
**>200 cell/µL, %**	26%	19.2%	25.4%	24.1%	25.6%	21.1%	25.3%	28.1%	32.6%	34.9%
**Less than 350 cell/µL CD4 at enrollment, %**	44.2%	34%	41.4%	42.1%	45.7%	40.8%	44.4%	46.4%	52.8%	54.5%
**AIDS Presenters, %**	12.1%	10.6%	14.2%	13.4%	17.7%	9.4%	11%	11.8%	13.6%	14.2%
**HCV serostatus at enrollment**										
** HCV positive, %**	19%	52.3%	34.6%	21.6%	17.1%	10.7%	9.6%	6.8%	6.5%	4.8%
**HBsAg serostatus at enrollment**										
**HBsAg positive**	5.7%	8%	7.1%	7.4%	5.8%	6.7%	5%	4.7%	3.9%	4.4%

CI, confidence interval; HE, heterosexual; MSM, men who have sex with men; PWID, people who inject drugs.

A significant rise in the proportion of people with CD4+ T cell count <350 cells/µL and <200 cells/µL at enrollment was observed, increasing from 34.0% to 26.0% in 1997–1998 to 54.5% and 34.9% in 2020–2022, respectively.

### Crude All-Cause and Cause-Specific Mortality Rates

**Figure 1.**
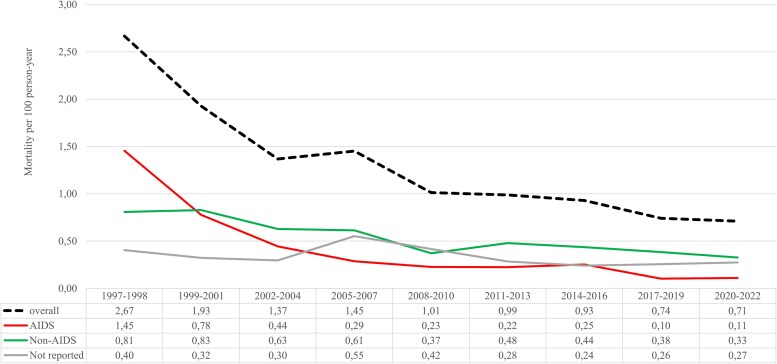
All cause and cause specific crude mortality according to calendar period.

Figure 1 shows the crude all-cause and cause-specific mortality rate per 100 person-years by calendar period, while Table 2 outlines the rate of death of the cohort. Overall, the crude mortality rate decreased progressively over time. The highest rates were observed during the earliest calendar periods, with 2.67 (95% CI 2.19–3.25) and 1.93 (95% CI 1.67–2.22) deaths per 100 person-years in 1997–1998 and 1999–2001, respectively. After 2010, mortality rates dropped below 1 per 100 person-years, reaching 0.74 (95% CI 0.65–0.84) and 0.71 (95% CI 0.63–0.80) in 2017–2019 and 2020–2022, respectively. Regarding cause-specific mortality, a significant drop was observed for AIDS-related mortality in the first two periods from 1.45 (95% CI 1.11–1.90) in 1997–1998 to 0.78 (95% CI 0.62–0.97) deaths per 100 person-years in 1999–2001. AIDS-related mortality continued to drop in the subsequent years, with the lowest mortality rate observed in the last 2 periods: 0.10 (95% CI 0.07–0.14) deaths per 100 person-years in 2017–2019 and 0.10 (95% CI 0.08–0.15) deaths per 100 person-years in 2020–2022, respectively.

**Table 2.** Crude All-cause and Cause Specific Mortality Rate per Calendar Period

	Calendar Period								
	1997–1998	1999–2001	2002–2004	2005–2007	2008–2010	2011–2013	2014–2016	2017–2019	2020–2022
**Number of deaths**	99	191	148	142	112	167	228	238	259
**100-person year**	37.11	99.05	108.19	97.81	110.57	169.10	245.49	320.94	364.53
**Overall crude mortality rate** **(95% CI)**	2.67 (2.19–3.25)	1.93 (1.67–2.22)	1.37 (1.16–1.61)	1.45 (1.23–1.71)	1.01 (0.84–1.22)	0.99 (0.85–1.15)	0.93 (0.82–1.06)	0.74 (0.65–0.84)	0.71 (0.63–0.80)
**Cause specific crude mortality** **Rate (95% CI)**									
** AIDS**	1.45 (1.11–1.90)	0.78 (0.62–0.907)	0.44 (0.33–0.59)	0.29 (0.20–0.41)	0.23 (0.15–0.33)	0.22 (0.16–0.31)	0.25 (0.20–0.32)	0.10 (0.07–0.14)	0.11 (0.08–0.15)
** NON-AIDS**	0.81 (0.57–1.16)	0.83 (0.67–1.03)	0.63 (0.50–0.80)	0.61 (0.48–0.79)	0.37 (0.27–0.50)	0.48 (0.39–0.60)	0.44 (0.36–0.53)	0.38 (0.32–0.46)	0.33 (0.27–0.39)
** Unknown**	0.40 (0.24–0.67)	0.32 (0.23–0.46)	0.30 (0.21–0.42)	0.55 (0.42–0.72)	0.42 (0.31–0.56)	0.28 (0.21–0.38)	0.24 (0.19–0.31)	0.26 (0.21–0.32)	0.27 (0.23–0.33)

CI, confidence interval.

### Adjusted All-Cause Mortality Rates

Table 3 presents adjusted mortality rate estimates across calendar periods, while relative risk ratios (RRs) are provided in Supplementary Table 1. In 1997–1998, the mortality rate was significantly higher in males than females (RR: 2.07, 95% CI 1.21–3.54), but both sexes experienced a parallel reduction in mortality over time. PWID had consistently higher mortality risks compared to those with HE risk factor. This risk was attenuated only during 2020–2022 [RR 1.28 (95% CI: 0.91–1.81)] when the mortality rates for PWID dropped to 0.62 (95% CI: 0.39–0.95) per 100 person-years from 1.21 (95% CI: 0.92–1.49) in 2017–2019.

**Table 3.** Adjusted Death Rate per 100 Person-year According to Different Exposure

	Calendar Period								
	1997–1998	1999–2001	2002–2004	2005–2007	2008–2010	2011–2013	2014–2016	2017–2019	2020–2022
**Overall^a^**	3.78 (3.03–4.54)	2.45 (2.10–2.79)	1.51 (1.27–1.76)	1.43 (1.20–1.67)	0.91 (0.74–1.08)	0.85 (0.72–.99)	0.76 (0.66–0.86)	0.56 (0.49–0.63)	0.48 (0.42–0.54)
**Sex assigned at birth^b^**									
**Male**	4.43 (3.47–5.39)	2.41 (2.00–2.82)	1.51 (1.22–1.81)	1.47 (1.19–1.76)	0.90 (0.70–1.10)	0.81 (0.66–0.96)	0.70 (0.59–0.81)	0.60 (0.51–0.68)	0.50 (0.43–0.57)
**Female**	2.14 (1.08–3.20)	2.50 (1.84–3.17)	1.50 (1.05–1.95)	1.33 (0.91–1.75)	0.93 (0.61–1.26)	0.97 (0.69–1.25)	0.93 (0.70–1.16)	0.43 (0.30–0.56)	0.42 (0.30–0.54)
**Place of birth^a^**									
**Non Italian**	7.60 (2.29–12.91)	3.53 (1.49–5.57)	1.56 (0.39–2.71)	2.13 (0.85–3.41)	0.99 (0.30–1.68)	1.22 (0.71–1.74)	1.08 (0.72–1.43)	0.58 (0.37–0.78)	0.50 (0.33–0.66)
**Italian**	3.64 (2.89–4.40)	2.40 (2.05–2.76)	1.51 (1.26–1.76)	1.39 (1.15–1.63)	0.90 (0.73–1.08)	0.81 (0.68–0.94)	0.71 (0.61–0.81)	0.55 (0.47–0.62)	0.47 (0.41–0.54)
**Age at enrollment^c^**									
**<45**	2.65 (2.09–3.20)	1.80 (1.51–2.08)	1.29 (1.04–1.52)	1.17 (0.91–1.43)	0.68 (0.47–0.88)	0.56 (0.40–0.71)	0.44 (0.32–0.52)	0.29 (0.21–0.38)	0.27 (0.19–0.32)
**>45**	3.06 (1.23–4.09)	2.87 (1.95–3.78)	1.73 (1.18–2.29)	2.13 (1.60–2.67)	1.50 (1.14–1.85)	1.50 (1.22–1.77)	1.44 (1.22–1.66)	1.15 (0.99–1.31)	1.03 (0.89–1.16)
**Mode of HIV acquisition^b^**									
**HE**	2.31 (1.29–3.34)	1.62 (1.15–2.09)	0.93 (0.62–1.24)	1.00 (0.69–1.31)	0.63 (0.41–0.85)	0.74 (0.55–0.93)	0.77 (0.62–0.92)	0.43 (0.33–0.52)	0.48 (0.39–0.57)
**MSM**	3.20 (1.61–4.77)	1.47 (0.87–2.08)	0.58 (0.25–0.91)	0.87 (0.49–1.26)	0.68 (0.40–0.95)	0.48 (0.31–0.65)	0.47 (0.34–0.60)	0.41 (0.31–0.60)	0.37 (0.29–0.45)
**PWID**	5.03 (3.69–6.67)	3.69 (3.00–4.38)	2.64 (2.01–3.20)	2.17 (1.65–2.68)	1.61 (1.17–2.04)	1.50 (1.12–1.87)	1.14 (0.84–1.43)	1.21 (0.92–1.49)	0.62 (0.43–0.80)
**Other**	6.05 (1.49–10.61)	2.86 (1.06–4.55)	2.14 (0.92–3.37)	2.47 (1.17–3.83)	0.67 (0.01–1.33)	1.21 (0.54–1.87)	1.01 (0.54–1.48)	0.71 (0.37–1.04)	0.67 (0.39–0.95)
**CD4 at enrollment^a^**									
**<200 cell/µL**	10.59 (7.73–13.45)	4.84 (3.76–5.92)	2.26 (1.60–2.93)	1.81 (1.21–2.40)	1.05 (0.65–1.46)	1.52 (1.15–1.89)	1.60 (1.30–1.90)	0.84 (0.66–1.02)	0.91 (0.74–1.08)
**200–349 cell/µL**	3.32 (1.50–5.13)	2.86 (1.89–3.83)	1.44 (0.83–2.05)	1.80 (1.12–2.47)	1.04 (0.60–1.49)	1.00 (0.65–1.34)	0.64 (0.42–0.86)	0.56 (0.39–0.74)	0.43 (0.30–0.57)
**350–499 cell/µL**	2.71 (1.37–4.04)	1.69 (1.06–2.31)	1.43 (0.91–1.96)	1.35 (0.84–1.86)	0.88 (0.51–1.25)	0.52 (0.29–0.75)	0.47 (0.30–0.65)	0.52 (0.37–0.68)	0.35 (0.23–0.46)
**>500 cell/µL**	1.09 (0.49–1.68)	1.37 (0.98–1.77)	1.20 (0.87–1.53)	1.20 (0.87–1.53)	0.81 (0.56–1.07)	0.62 (0.44–0.81)	0.46 (0.33–0.59)	0.43 (0.32–0.54)	0.31 (0.22–0.39)
**CD4 < 350 cell/µL at enrollment^a^**									
**No**	1.64 (1.04–2.24)	1.49 (1.15–1.82)	1.28 (1.00–1.56)	1.24 (0.97–1.52)	0.83 (0.62–1.04)	0.59 (0.44–0.73)	0.46 (0.36–0.57)	0.46 (0.37–0.54)	0.32 (0.25–0.39)
**Yes**	7.51 (5.69–9.31)	4.03 (3.28–4.77)	1.91 (1.45–2.37)	1.79 (1.35–2.23)	1.04 (0.74–1.34)	1.28 (1.03–1.54)	1.19 (0.99–1.38)	0.71 (0.59–0.84)	0.71 (0.59–0.82)
**AIDS presenter^a^**									
**No**	2.12 (1.53–2.70)	1.90 (1.56–2.22)	1.30 (1.07–1.55)	1.33 (1.08–1.57)	0.85 (0.67–1.03)	0.68 (0.55–0.80)	0.58 (0.48–0.67)	0.52 (0.45–0.59)	0.43 (0.37–0.49)
**Yes**	16.25 (11.44–21.05)	6.35 (4.66–8.04)	3.18 (2.07–4.28)	2.40 (1.46–3.33)	1.60 (0.92–2.28)	2.45 (1.78–3.15)	2.40 (1.85–2.96)	1.08 (0.78–1.39)	1.08 (0.81–1.36)
**HCV at enrollment^a^**									
**No**	3.20 (2.22–4.18)	1.95 (1.52–2.38)	0.93 (0.68–1.18)	0.96 (0.71–1.21)	0.65 (0.47–0.82)	0.65 (0.52–0.79)	0.69 (0.58–0.79)	0.46 (0.38–0.053)	0.43 (0.37–0.50)
**Yes**	4.45 (3.28–5.62)	3.04 (2.47–3.63)	2.33 (1.85–2.80)	2.14 (1.68–2.61)	1.41 (1.05–1.77)	1.37 (1.05–1.69)	0.94 (0.71–1.17)	0.94 (0.73–1.16)	0.65 (0.48–0.82)
**HBsAg positivity at enrollment^a^**									
**No**	3.77 (2.98–4.55)	2.35 (2.00–2.71)	1.50 (1.24–1.75)	1.43 (1.18–1.68)	0.90 (0.73–1.08)	0.82 (0.69–0.96)	0.76 (0.66–0.86)	0.55 (0.48–0.63)	0.47 (0.40–0.53)
**Yes**	3.92 (1.33–6.50)	3.42 (1.96–4.89)	1.67 (0.75–2.60)	1.45 (0.59–2.32)	1.02 (0.39–1.66)	1.27 (0.67–1.87)	0.70 (0.33–1.07)	0.64 (0.33–0.95)	0.73 (0.43–1.02)

HE, heterosexual; MSM, men who have sex with men; PWID, people who inject drugs.

^a^Adjusted for sex assigned at birth and the mean age of the calendar period.

^b^Adjusted for the mean age of the calendar period.

^c^Adjusted for sex assigned at birth.

Mortality rates remained significantly higher for PWH with AIDS at enrollment compared to those without with a drop in mortality rates observed in 2017–2019 and 2020–2022 [1.08 (95% CI 0.78–1.09) and 1.08 (95% CI 0.81–1.36) per 100 person-years, respectively].

In PWH with a positive serology for HCV at enrollment, mortality RRs remained elevated throughout the study period with a persistent 50% higher risk of death compared to those who had negative anti-HCV antibodies (RR 1.50, 95% CI: 1.12–1.99) in the last calendar period (2020–2022). Adjusted all-cause mortality rates, accounting for age and sex assigned at birth, are illustrated in Supplementary Figure 2, and adjusted mortality rate according to different characteristics at the time of enrollment is reported in Supplementary Figures 3–10.

### Causes of Death and Characteristics at the Time of Death

**Figure 2.**
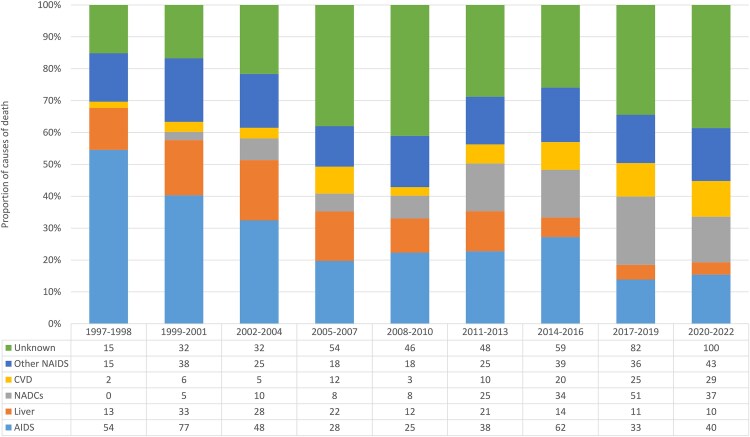
Causes of death according to calendar period. NAIDS, non-AIDS; CVD, cardiovascular disease; NADC, non-AIDS defining cancer.

Figure 2 depicts the distribution of cause-specific deaths. AIDS was the leading cause of death in early calendar periods, declining significantly from 54.5% in 1997–1998 to 19.7% in 2005–2007, with 13.9% and 15.4% in 2017–2019 and 2020–2022, respectively. Liver-related deaths were prominent from 1999 to 2007, while non-AIDS cancers emerged as a leading cause from 2011 to 2013 (15%), accounting for up to 21% of deaths in 2017–2019. Cardiovascular deaths increased in the last two calendar periods (2017–2019 and 2020–2022: 10.5% and 11.2%, respectively), while deaths from other non-AIDS-related causes remained stable over time. Unknown causes accounted for approximately one-third of deaths, with higher proportions observed in recent years.

The mean age at death increased over time, from 38 years (95% CI: 36–40) in 1997–1998 to 58 years (95% CI: 57–59) in 2020–2022 (Supplementary Table 2). The mean age at death by cause is depicted in Supplementary Figure 1^1^).

## DISCUSSION

In our Italian multicenter cohort, consisting of 17 006 PWH, a significant progressive decline in both all-cause and AIDS-specific mortality was observed. This trend primarily reflects the adoption of the first ART in the earlier periods of the study (1997–1998), the following gradual expansion and improvement of ART coverage, and the implementation of the “ART for all” approach after 2016, ensuring treatment for all newly diagnosed individuals regardless of disease stage or CD4+ T cell count [2, 20].

A series of social changes in the 1980s and 1990s led to the widespread use of intravenous heroin among young people in Italy [13], influencing shifts in age and risk factors for HIV acquisition. In the late 1990s, the cohort predominantly consisted of young individuals, with a mean age of 35.6 years, but the median enrollment age increased to 41.7 years after 2020. Initially, a high proportion of PWID (47.1%) at enrollment was observed, which later declined to 4.6% [13]. This reduction in injection-related transmission parallels a decline in chronic HCV infection prevalence. These trends align with Western Europe [21], though the proportion of PWID and individuals tested positive for HCV in this cohort was higher than in other multicenter HIV studies during the same period [4, 7].

As the proportion of PWID declined, the proportion of MSM among newly diagnosed PWH increased from 17% to 48.3% [4, 22]. Greater societal acceptance and reduced stigma may have contributed to more accurate self-reporting of sexual behaviors in MSM [23]. The initial 17% figure may have been underestimated due to stigma-driven underreporting from the 1960s to the 2000s. Although less pronounced than in MSM, the proportion of HE PWH also increased (from 32.1% to 38.2%), a trend that should be considered alongside the rising number of migrants among newly diagnosed PWH, consistent with official Italian reports [16].

Late presentation among PWH remains a major epidemiological challenge across Europe [24], with Italy reporting the high rates in Western Europe, significantly impacting survival probability [16, 25]. In recent years, the proportion of individuals presenting with AIDS (up to 14.1% in 2020–2022) has increased [15]. These trends align with official European reports [24]. The high frequency of late presentation likely contributes to the observed plateau in AIDS-related mortality (0.11 deaths per 100 person-years in 2020–2022).

The crude all-cause mortality rates demonstrated a significant decline over time, decreasing from 2.67 deaths per 100 person-years in 1997–1998 to 0.71 in 2020–2022. This trend remained consistent even after adjusting for age and sex reflecting the successful implementation of ART that improved all the PWH health outcomes [3, 4, 7, 9–11, 26]. The examining mortality rates over time suggest that our cohort reflects the changes in treatment guidelines. A marked improvement in mortality rates was observed in the late 1990s, coinciding with one of the first milestones in the HIV field: the introduction of protease inhibitors [9 ]. A further significant reduction occurred when the treatment threshold was raised up to 350 CD4 cells/µL [27]. Finally, an additional and substantial decrease was obtained after 2016, following the introduction of universal ART, endorsed by results of START study [2], and its subsequent adoption into Italian [28] and international guidelines [20].

The impact of ART, particularly early initiation, is evident in cause-specific mortality trends. AIDS-related deaths, the primary cause of mortality in the early years, declined from 54.5% in 1997–1998 to 15.4% in 2020–2022, reflecting global trends driven by ART expansion and earlier HIV diagnosis [6–9]. In contrast, non-AIDS-related causes have become the leading contributors to mortality. Liver-related deaths were prominent between 1999 and 2007 but have since declined due to three factors: the introduction of direct-acting antivirals reducing chronic HCV infection [29], the natural progression of untreated HCV leading to end-stage liver disease and subsequent mortality among early cohort enrollees, and the introduction of HBV vaccination in the 1990s, which reduced HBV prevalence by targeting birth cohorts from 1980 onward and at-risk groups. The mortality rate adjusted for age and sex did not show significant differences across calendar periods in individuals with or without HBsAg positivity. Nevertheless, given the small number of HBsAg positive individuals, the analysis appears to be underpowered to detect meaningful differences in mortality between HBsAg positive and negative participants.

The extended life expectancy granted by effective ART has led to an increase in non-AIDS-related deaths, primarily cancers and cardiovascular diseases, now the leading causes of mortality [4, 7, 8]. Reflecting global aging patterns in PWH cohorts, the mean age at death rose from 38 years in 1997–1998 to 58 years in 2020–2022 [4, 7, 8]. The rise in deaths classified as “unknown” is expected, as more people now die outside hospital settings, complicating cause-of-death determination [4, 7, 8].

AIDS-related deaths in recent years occur at a younger mean age (53 years) compared to non-AIDS-related deaths (61 years). Mortality remains significantly higher among those with lower CD4+ T cell counts (<350 cells/µL) and those presenting with AIDS at enrollment, with relative risks of 2.50 and 2.22, respectively, compared to those without such conditions [7, 30]. Notably, mortality rates in people presenting with AIDS have remained unchanged since 2016, suggesting that ART alone may be insufficient in late-stage HIV infection. Only earlier diagnosis, alongside effective ART, can improve outcomes. While overall mortality reduction is largely driven by fewer AIDS-related deaths, these still accounted for 15.4% of all deaths in 2020–2022—a proportion significantly higher than in the Swiss cohort [8] but consistent with findings from the DAD multicohort study [4] and the RESPOND study [11].

Adjusted all-cause mortality rates initially showed notable disparities by sex and age, with males exhibiting a higher risk of death than females (RR: 2.07) in 1997–1998. However, this gap diminished over time, with no significant differences observed in later periods, aligning with global findings that ART has improved survival for both sexes [3, 4].

A significant mortality disparity was also observed between individuals born in Italy and those born abroad in the first calendar period (1997–1998), with Italians having a lower risk of death (RR: 0.48). Over time, this gap narrowed, with mortality risks becoming comparable in the most recent periods. Mortality ascertainment through official registries is less reliable for foreign-born individuals, particularly those with temporary or irregular status, potentially leading to an underestimation of deaths in this group.

### Limitations

Our study presents several limitations. First, although Icona is the largest cohort of newly diagnosed PWH in Italy, it does not cover the entire Italian territory, with some entire region completely missing (ie, Calabria, Valle D’Aosta). Second, PWH at higher risk of short-term mortality, particularly those who are hospitalized and critically ill (unable to provide a written informed consent), may be less likely to be enrolled in the Icona cohort. This could introduce a selection bias toward persons with a better prognosis. Third, peripheral, small centers are underrepresented, meaning our findings may not be directly generalizable to settings not included in the Icona cohort. Fourth, the assignment of causes of death might have introduced inaccuracies relative to the true causes of death. Fifth, gender information would have strengthened our manuscript. However, gender data collection was only implemented in the most recent year in the ICONA cohort, and such information was unavailable for the majority of PWH included in the present analysis. Therefore, we opted to use sex assigned at birth as the exposure variable to ensure consistency across different calendar years. Sixth, not all ICONA centers participated in the study, as some were unable to ensure adequate participant follow-up or assess people vital status through official regional and national administrative registries. In the end, it must be acknowledged that the ICONA cohort has contributed to prior multicohort studies that assessed mortality and causes of death. (D:A:D, RESPOND, ART-CC [11 , 31 , 32 ]). Nevertheless the current study presents distinct and novel features. Unlike previous analyses, it exclusively includes ART-naïve individuals at baseline [11], offering a more homogeneous population. Additionally, it features enhanced cause-of-death classification via linkage to regional and national administrative databases, improving data completeness and reliability. This methodological refinement enabled the inclusion of 17 006 individuals, with 1584 deaths (9.31%), surpassing earlier sample sizes [31]. Though overlapping with cohorts used in recent studies [11, 31, 32], the current analysis uniquely incorporates deaths up to 2022. This extended follow-up allows for an assessment of mortality trends during the early COVID-19 pandemic. Despite these limitations, our study has the strength of including a large number of PWH with baseline characteristics similar to those reported in the official Italian data for newly diagnosed PWH in Italy [16].

## CONCLUSIONS

Our study highlights the success of ART in significantly reducing all-cause mortality in PWH with a great contribution in this reduction made by a significant decrease in AIDS-related mortality. People with a positive HCV serology and with AIDS at HIV diagnosis continue to show a high risk of death even in the more recent years. Reliable surveillance data on mortality and causes of death are essential, as mortality serves as a critical indicator to assess population levels interventions in a country, such as Italy, characterized by the highest proportion of PWH presenting late in Europe.

## Supplementary Material

ofaf455_Supplementary_Data

## Supplementary Data

Supplementary materials are available at *Open Forum Infectious Diseases* online. Consisting of data provided by the authors to benefit the reader, the posted materials are not copyedited and are the sole responsibility of the authors, so questions or comments should be addressed to the corresponding author.
