# Supplementary material for: All-cause and Cause-specific Mortality in People With HIV in Italy in 1997–2022: Data From the Icona Cohort
Source: Open Forum Infect Dis. 2025 Aug 2;12(8):ofaf455. doi: 10.1093/ofid/ofaf455 (PMC12343120; doi:10.1093/ofid/ofaf455)
Supplement: ofaf455_Supplementary_Data [file ofaf455_supplementary_data.docx]

**Supplementary Table 1. Adjusted risk ratio of death according to different exposure.**

|  | Calendar period | | | | | | | | |
| --- | --- | --- | --- | --- | --- | --- | --- | --- | --- |
| Adjusted Risk Ratio (95%CI) | **1997-1998** | **1999-2001** | **2002-2004** | **2005-2007** | **2008-2010** | **2011-2013** | **2014-2016** | **2017-2019** | **2020-2022** |
| Sex assigned at birth^†^ |  |  |  |  |  |  |  |  |  |
| M *vs* F | 2.07  (1.21-3.54) | 0.96  (0.70-1.32) | 1.01  (0.71-1.44) | 1.11  (0.76-1.61) | 0.97  (0.64-1.46) | 0.83  (0.59-1.17) | 0.75  (0.57-1.01) | 1.39  (0.99-1.94) | 1.19  (0.87-1.63) |
| Age at enrolment^⸸^ |  |  |  |  |  |  |  |  |  |
| >45 *vs* <45 | 1.15  (0.61-2.18) | 1.59  (1.11-2.28) | 1.34  (0.93-1.95) | 1.82  (1.31-2.54) | 2.20  (1.51-3.22) | 2.69  (1.93-3.74) | 3.30  (2.44-4.48) | 3.94  (2.84-5.45) | 3.80  (2.73-5.30) |
| Place of birth^*^ |  |  |  |  |  |  |  |  |  |
| Italian vs non Italian | 0.48  (0.23-0.99) | 0.68  (0.37-1.23) | 0.97  (0.45-2.09) | 0.65  (0.39-1.22) | 0.91  (0.44-1.87) | 0.66  (0.42-1.04) | 0.66  (0.45-0.94) | 0.95  (0.65-1.39) | 0.95  (0.66-1.35) |
| Mode of HIV acquisition^†^ |  |  |  |  |  |  |  |  |  |
| MSM vs HE | 1.38  (0.71-2.67) | 0.91  (0.55-1.50) | 0.62  (0.33-1.21) | 0.87  (0.51-1.50) | 1.08  (0.63-1.84) | 0.65  (0.42-1.00) | 0.61  (0.44-0.86) | 0.96  (0.69-1.33) | 0.77  (0.58-1.03) |
| PWID vs HE | 2.17  (1.30-3.63) | 2.28  (1.61-3.22) | 2.84  (1.91-4.20) | 2.16  (1.46-3.20) | 2.57  (1.65-3.99) | 2.01  (1.41-2.88) | 1.47  (1.06-2.03) | 2.81  (2.05-3.86) | 1.28  (0.91-1.81) |
| Other vs HE | 2.61  (1.09-6.27) | 1.76  (0.88-3.53) | 2.31  (1.19-4.45) | 2.47  (1.31-4.64) | 1.07  (0.38-3.05) | 1.62  (0.89-2.97) | 1.31  (0.79-2.17) | 1.65  (0.98-2.76) | 1.40  (0.89-2.19) |
| CD4 at enrollment (cell/µL) ^*^ |  |  |  |  |  |  |  |  |  |
| 200-350 *vs* <200 | 0.31  (0.17-0.58) | 0.59  (0.39-0.89) | 0.64  (0.38-1.06) | 0.99  (0.61-1.63) | 0.99  (0.55-1.76) | 0.66  (0.43-0.99) | 0.40  (0.27-0.59) | 0.67  (0.47-0.97) | 0.48  (0.33-0.68) |
| 350-500 *vs* <200 | 0.26  (0.14-0.45) | 0.34  (0.22-0.54) | 0.63  (0.40-1.01) | 0.74  (0.45-1.23) | 0.83  (0.47-1.47) | 0.34  (0.21-0.56) | 0.30  (0.20-0.45) | 0.62  (0.43-0.89) | 0.38  (0.26-0.56) |
| >500 *vs* <200 | 0.11  (0.06-0.19) | 0.28  (0.20-0.40) | 0.53  (0.35-0.79) | 0.66  (0.43-1.02) | 0.78  (0.47-1.27) | 0.41  (0.28-0.60) | 0.29  (0.20-0.40) | 0.51  (0.37-0.71) | 0.33  (0.24-0.47) |
| CD4 <350 cell/µL at presentation^*^ |  |  |  |  |  |  |  |  |  |
| Yes vs no | 4.57  (2.95-7.08) | 2.71  (2.02-3.63) | 1.49  (1.08-2.07) | 1.44  (1.03-2.01) | 1.24  (0.85-1.82) | 2.18  (1.60-2.99) | 2.57  (1.95-3.39) | 1.56  (1.21-2.03) | 2.22  (1.71-2.88) |
| AIDS presentation^*^ |  |  |  |  |  |  |  |  |  |
| Yes vs no | 7.67  (5.11-11.50) | 3.34  (2.44-4.59) | 2.42  (1.64-3.60) | 1.80  (1.17-2.77) | 1.88  (1.17-3.02) | 3.64  (0.61-5.08) | 4.17  (3.16-5.51) | 2.08  (1.52-2.83) | 2.50  (1.89-3.31) |
| HCV postive^*^ |  |  |  |  |  |  |  |  |  |
| Yes vs no | 1.39  (0.93-2.08) | 1.56  (1.17-2.09) | 2.51  (1.79-3.52) | 2.24  (1.60-3.14) | 2.18  (1.50-3.17) | 2.10  (1.54-2.86) | 1.37  (1.03-1.83) | 2.07  (1.58-2.71) | 1.50  (1.12-1.99) |
| HBsAg positive* |  |  |  |  |  |  |  |  |  |
| Yes vs no | 1.04  (0.52-2.08) | 1.45  (0.92-2.29) | 1.11  (0.62-1.99) | 1.01  (0.55-1.89) | 1.13  (0.59-2.17) | 1.55  (0.94-2.53) | 0.91  (0.53-1.58) | 1.16  (0.71-1.89) | 1.55  (1.01-2.38) |
| List HE, heterosexual; MSM, men who have sex with men; PWID, people who inject drugs.  ^†^Adjusted for the mean age of the calendar period  ^⸸^Adjusted for sex assigned at birth  ^*^ Adjusted for sex assigned at birth and the mean age of the calendar period | | | | | | | | | |

**Supplementary Table 2. Mean age at death according to different causes of death and calendar period.**

| Characteristics | Calendar period | | | | | | | | |
| --- | --- | --- | --- | --- | --- | --- | --- | --- | --- |
|  | **1997-1998** | **1999-2001** | **2002-2004** | **2005-2007** | **2008-2010** | **2011-2013** | **2014-2016** | **2017-2019** | **2020-2022** |
| Age, mean (95% CI) |  |  |  |  |  |  |  |  |  |
| Overall | 38 (36-40) | 40 (38-42) | 42 (40-44) | 45 (44-47) | 48 (46-50) | 50 (48-51) | 54 (53-55) | 55 (54-56) | 58 (57-59) |
| AIDS | 39 (36-41) | 41 (39-43) | 42 (39-45) | 45 (41-49) | 47 (43-51) | 48 (44-51) | 52 (49-54) | 50 (47-54) | 53 (50-56) |
| Non AIDS | 36 (32-40) | 38 (34-42) | 43 (41-46) | 47 (44-50) | 48 (44-51) | 51 (49-54) | 56 (54-58) | 58 (56-60) | 61 (59-63) |
| Unknown | 38 (33-44) | 38 (35-42) | 41 (37-44) | 44 (41-47) | 48 (45-51) | 48 (45-51) | 52 (49-56) | 53 (51-55) | 56 (54-58) |

Supplementary Figure 1. Cohort **enrollment** flow chart.

Supplementary Figure 2. Ange and sex adjusted mortality rate according to calendar period.

Supplementary Figure 3. Mortality rate according to different characteristics at **enrollment**-Age.


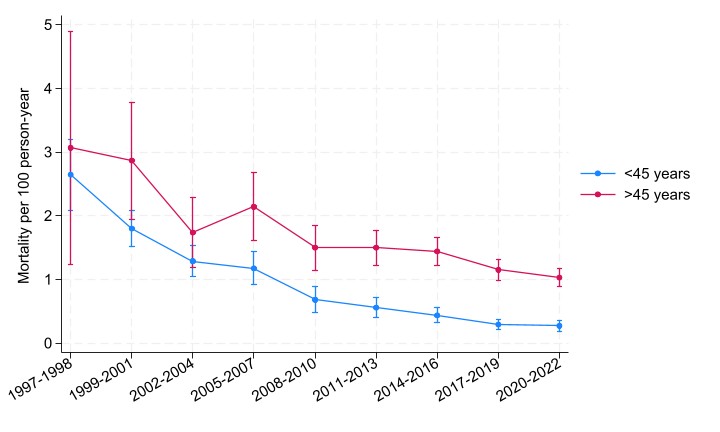


Supplementary Figure 4. Mortality rate according to different characteristics at **enrollment**-Biological sex.


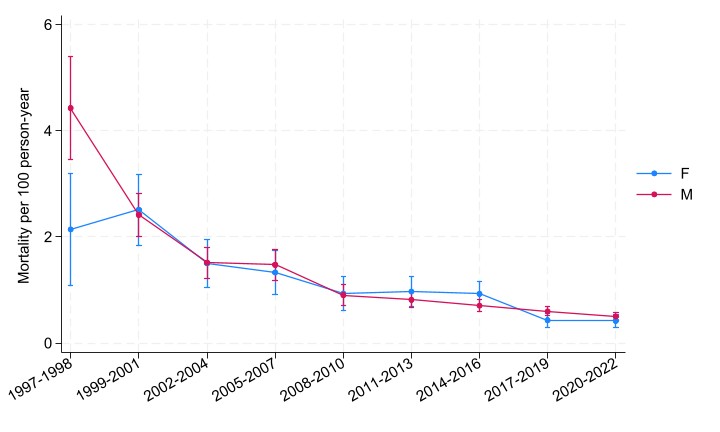


Supplementary Figure 5. Mortality rate according to different characteristics at **enrollment**-Country of birth.


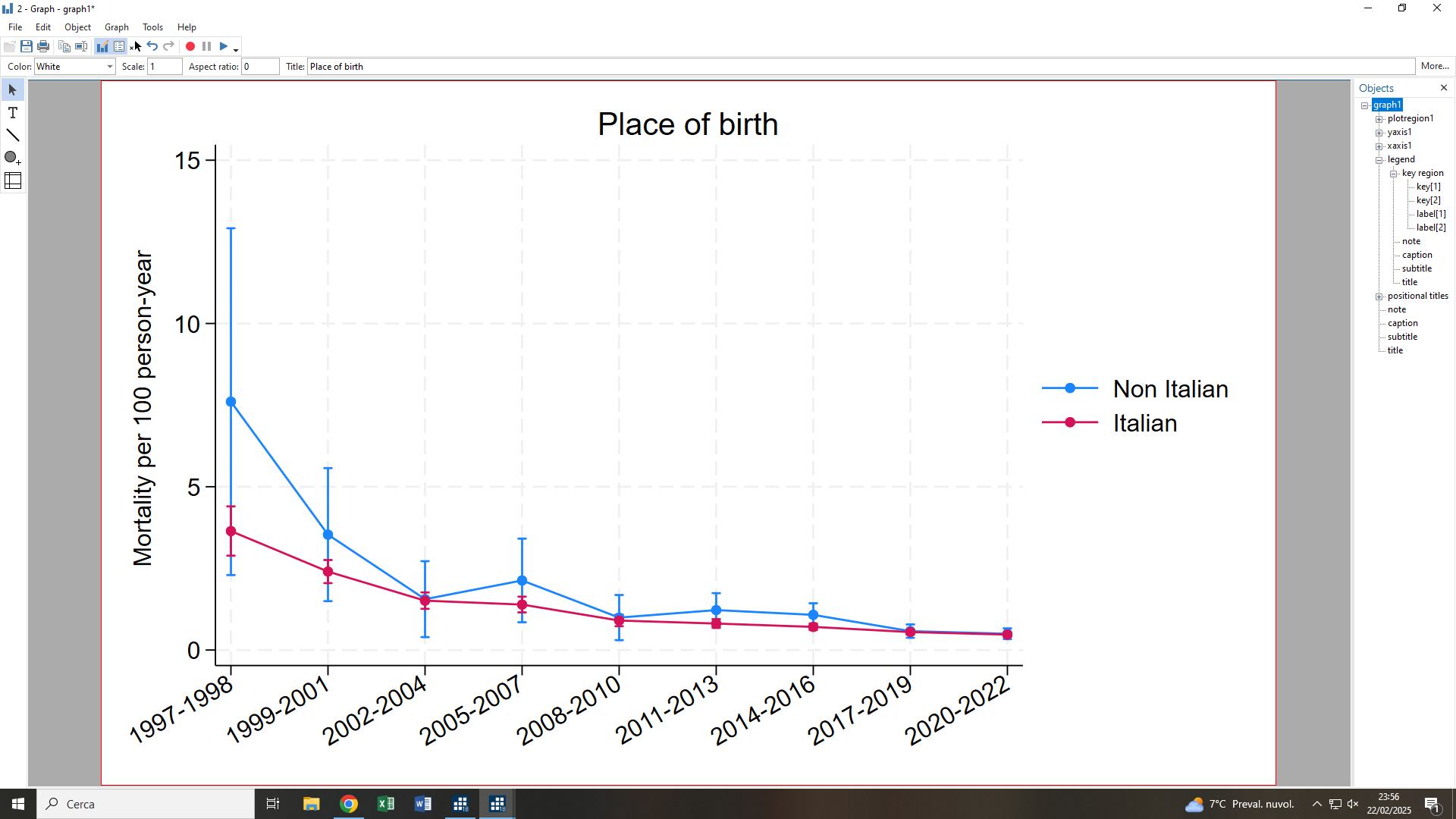


Supplementary Figure 6. Mortality rate according to different characteristics at **enrollment**-CD4 cell count.


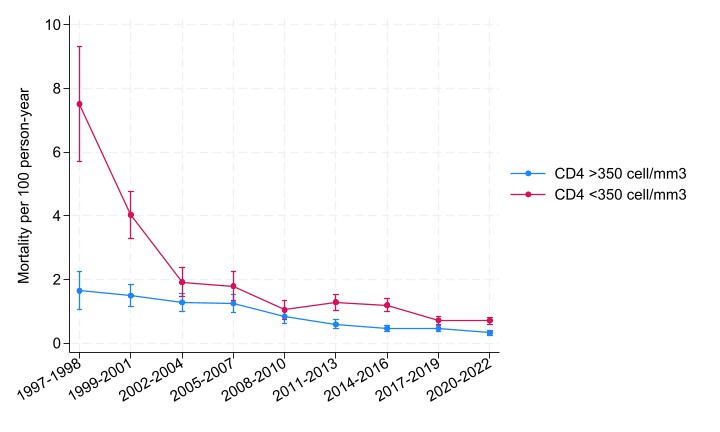


Supplementary Figure 7. Mortality rate according to different characteristics at **enrollment**-AIDS.


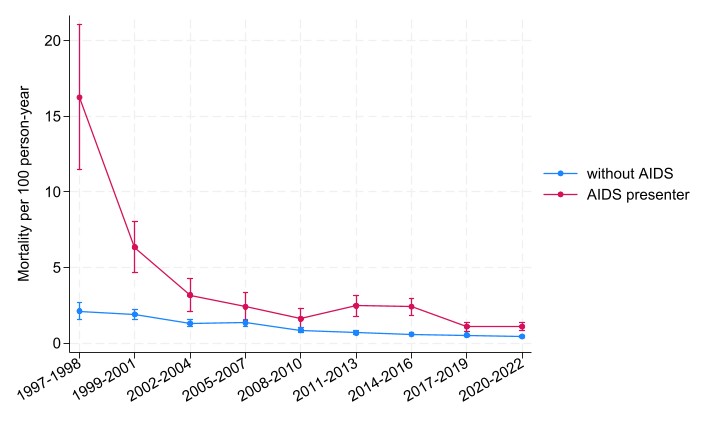


Supplementary Figure 8. Mortality rate according to different characteristics at **enrollment**-HCV.


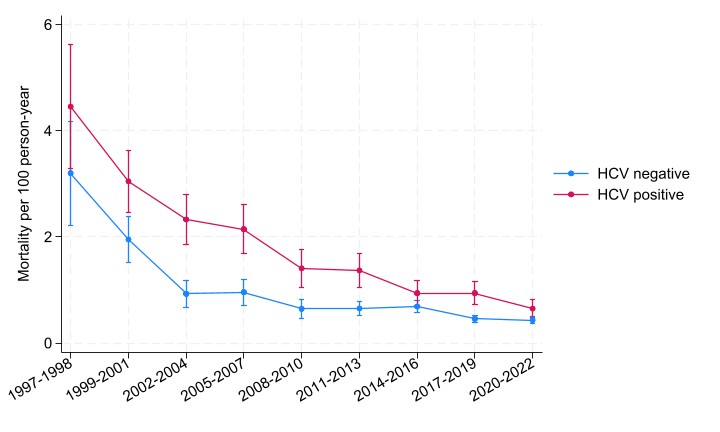


Supplementary Figure 9. Mortality rate according to different characteristics at **enrollment**-Mode of HIV acquisition.

Supplementary Figure 10. Mortality rate according to different characteristics at **enrollment**-HBsAg positivity.


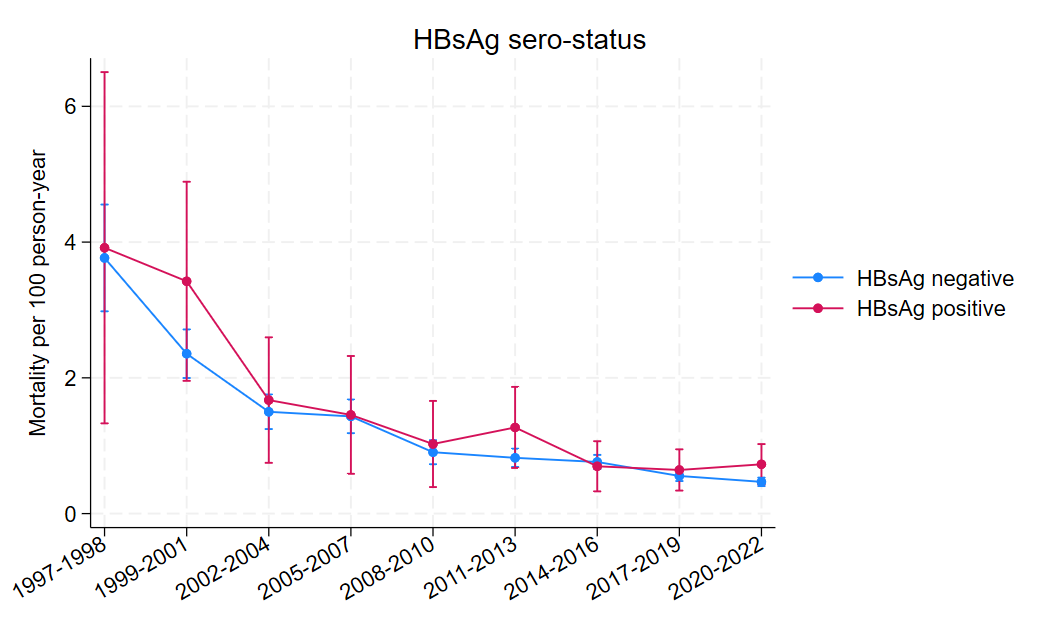


Supplementary Figure 11. Mean age at the time of death (all cause and cause specific) according to different calendar periods.
